# Handy EKG: A Low-Cost Electrocardiograph for Primary Care

**DOI:** 10.7759/cureus.48563

**Published:** 2023-11-09

**Authors:** Jhiamluka Solano, Alejandro J Calderón, Servio Paguada, Óscar Hernández, Erick Vladimir Reyes Marín, Hana Sandoval, Kellyn Funes, Raúl-José Palma-Mendoza

**Affiliations:** 1 Cardiology, Salford Royal NHS Foundation Trust, Manchester, GBR; 2 Department of Systems Engineering, Universidad Nacional Autónoma de Honduras, Tegucigalpa, HND; 3 General Medicine, Instituto Hondureño de Seguridad Social, Tegucigalpa, HND

**Keywords:** cardiology devices, machine learning models, preventive cardiology, primary care, electrocardiograph

## Abstract

Background

Cardiovascular diseases constitute the majority of noncommunicable disease deaths worldwide. In Honduras, cardiovascular diseases represent the fifth cause of death among individuals aged 45 to 49 years, while 20% of emergency room visits are due to cerebrovascular events, heart failure, and acute myocardial infarction.

Methodology

A low-cost three-lead electrocardiograph (ECG) (Handy EKG) was designed and manufactured for primary care. The device is supported by Bluetooth connectivity and machine learning. Device readings were collected from volunteers and compared to those obtained with a conventional 12-lead ECG.

Results

The device provided readings of lead one of a TDOU model CMS600G 12-lead ECG to monitor and diagnose bradyarrhythmia and tachyarrhythmias. Overall, 96% (49) of the readings showed a similarity in morphology, amplitude, and duration of waves, segments, and complexes compared to a 12-lead ECG. The device showed potential for application in primary care and intrahospital settings due to its continuous monitoring capabilities, portability, and possible connectivity with mobile devices.

Conclusions

The results indicate that the designed platform is safe, offers good quality in its operation at all levels, and provides ECG results equivalent to those of a conventional ECG in most cases (considering only one lead).

## Introduction

Noncommunicable diseases (NCDs) are long-lasting and result from genetic, physiological, environmental, and behavioral factors. Among the main types of NCDs, cardiovascular diseases (such as heart attacks and strokes) constitute the majority of NCD-related deaths (17.7 million each year) [1]. The economic burden is also important, with annual costs approximating $3.76 trillion in low- and middle-income countries. Developing countries face an enormous public health challenge due to the significant increase in these diseases in recent decades. In 2013, 20,137 deaths from NCDs were reported in Central America among individuals aged 65 to 74 years, with a mortality rate per 100,000 inhabitants of 1,326.25 [2].

In Honduras, cardiovascular diseases represent the fifth cause of death among individuals aged 45 to 49 years, with 20% of emergency room visits caused by cerebrovascular events, heart failure, and acute myocardial infarction. Additionally, 47.6% of the population is obese, 22.6% is hypertensive, and 7.2% have diabetes. Nonetheless, there are no available statistics on the annual mortality rates [3].

Diagnostic and screening tools such as the electrocardiogram (ECG) for early detection of cardiovascular diseases can significantly help reduce their morbidity. The Declaration of Alma-Ata [4] has recognized the implementation of ECGs in primary care as a practical tool to improve the quality of care. However, most first- and second-level health establishments in Honduras currently lack these tools due to funding limitations. Therefore, we developed a study to design a technological platform and a low-cost three-lead ECG that would be capable of providing results comparable to those of a conventional 12-lead ECG, provide an automatic preliminary diagnosis, and allow for essential data collection of factors associated with cardiovascular diseases in Honduras.

## Materials and methods

This research was developed in two general stages, namely, platform design and field validation. Both are described in more detail below.

Platform design stage

During this stage, the hardware design was performed by comparing microcontrollers and three-dimensional (3D) printing of the cases used in the prototypes. Subsequently, the embedded software that would enable the device to communicate between the different hardware elements was designed. Once the prototype’s functional stability was established, a mobile application was programmed and designed to provide portability to the prototype.

A web platform was designed to host the reference database to be accessible via the mobile application. Finally, during the validation stage, an automatic learning model was designed to allow the device to learn from each reading and provide a possible automatic diagnosis to the user.

Field validation stage

Pilot tests were conducted among volunteers without any previous clinical diagnosis of heart disease to adjust the software. During this stage, a conventional TDOU model CMS600G 12-lead ECG was used to make initial comparisons between the readings of both devices. Informed consent was obtained from the participants, and 10 tests were performed among patients between 20 and 60 years old with the conventional ECG and the prototype. Subsequently, several adjustments were made to the mobile and web applications to improve tracing readability, waiting times, and minor bug fixes.

Once the initial test was completed, data were collected from 51 healthy volunteers without any diagnosis of heart disease. These tests were performed among employees and visitors of the Honduran Medical College. Readings were taken with both the conventional ECG and the prototype for each volunteer. During the reading process, the medical research team offered the participants an interpretation of the results and medical guidance if necessary.

The results were analyzed by calculating central tendency and standard deviation measures and comparing the duration, amplitude, and morphology of the different ECG waves, complexes, and relevant segments on the readings from both the conventional ECG and the prototype. The morphology of the relevant complexes and segments was interpreted, classifying them as normal or abnormal and confirming whether the readings between devices were similar.

This project was reviewed and approved by the Directorate of Scientific Research and Postgraduate Studies of the National Autonomous University of Honduras (UNAH), which awarded a research grant to cover the costs of prototype design, creation, and validation.

## Results

Platform design stage

Phase 1. Hardware Design

**Figure 1:**
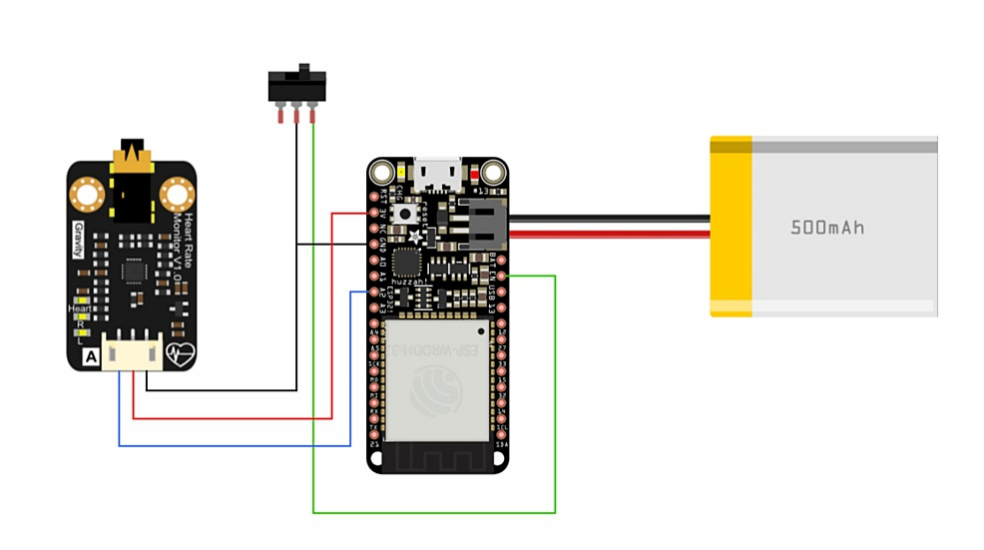
Device connection diagram.

**Figure 2:**
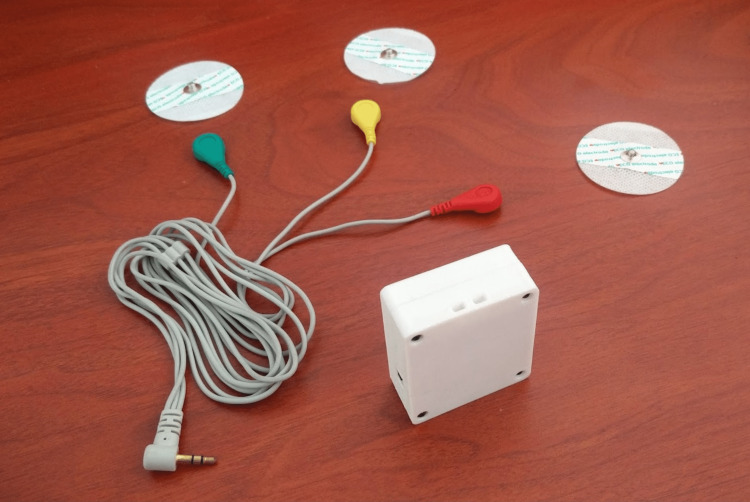
Final prototype.

Different tests were conducted with microcontroller evaluation boards to identify the most suitable ones during the design stage. The main component was an evaluation board based on the ESP32 chip, which provides a 240 MHz Tensilica LX6 microcontroller, Wi-Fi, and Bluetooth connectivity [5]. A module based on Analog’s AD8232 chip was used to capture the heart’s electrical signals and connect to one of the analog inputs on the main board [6]. To provide the device with autonomy and portability, a 500 mAh lithium-ion polymer (LiPo) battery was included in the design, as shown in Figure 1. Figure 2 shows the manufactured casing with 3D printing. The total cost of the hardware was US$15.

Phase 2. Embedded Software Design

Embedded software was used to establish communication between the device and the cell phone through the hardware application based on ESP32. This application is in charge of executing all the functionality of the device according to the commands sent from the cell phone. Communication between the device and the cell phone is done through a Bluetooth Low-Energy (BLE)-type connection. A Universal Asynchronous Receiver Transmitter channel was created over the BLE link using the service defined by Nordic Semiconductors [7].

Phase 3. Design of the Mobile Application

A mobile application was designed to allow users to perform the following functionalities: patient management, administration of medical appointments per patient, visualization, and downloading the electrocardiogram. The application has other secondary functionalities, such as configuration management, security, and a console to perform communication tests with the device.

**Figure 3:**
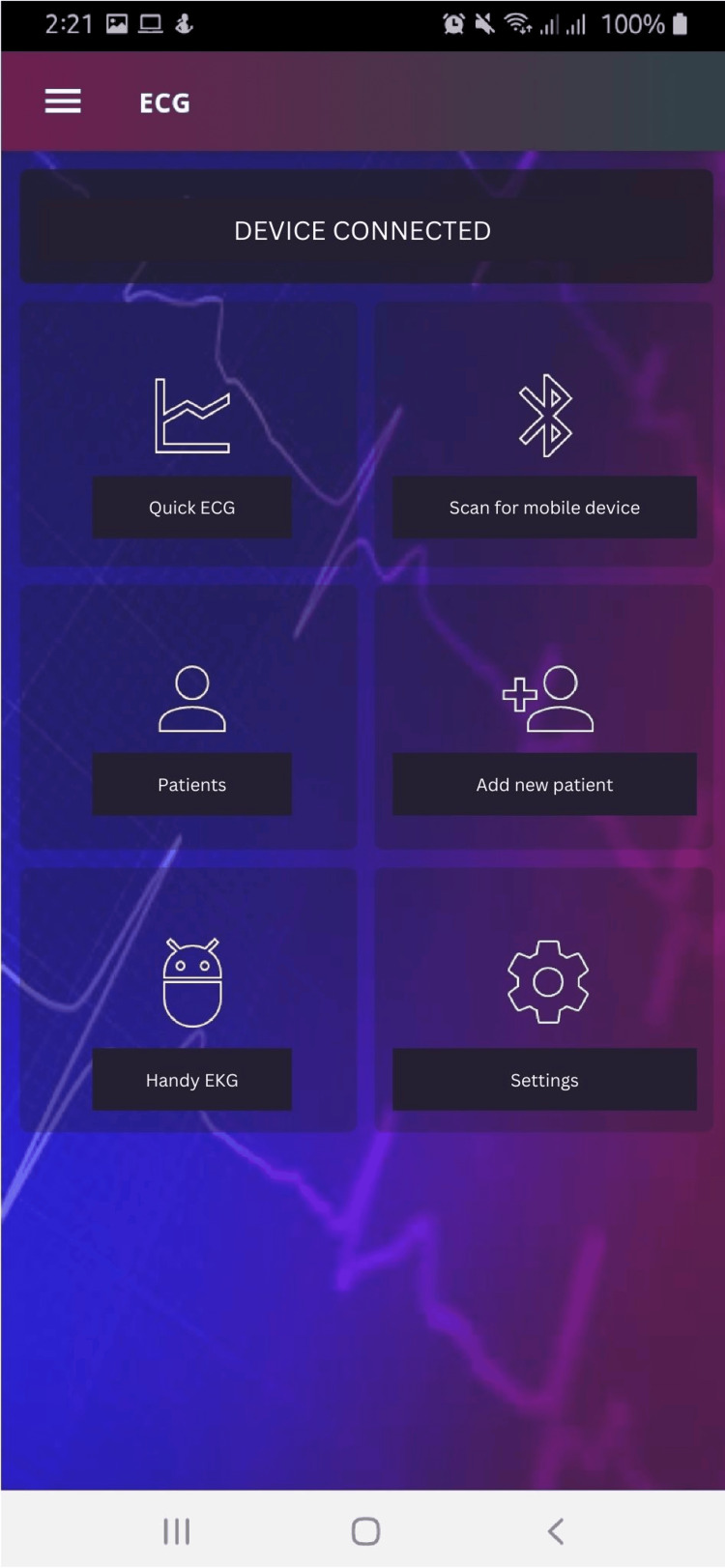
Mobile application interface.

**Figure 4:**
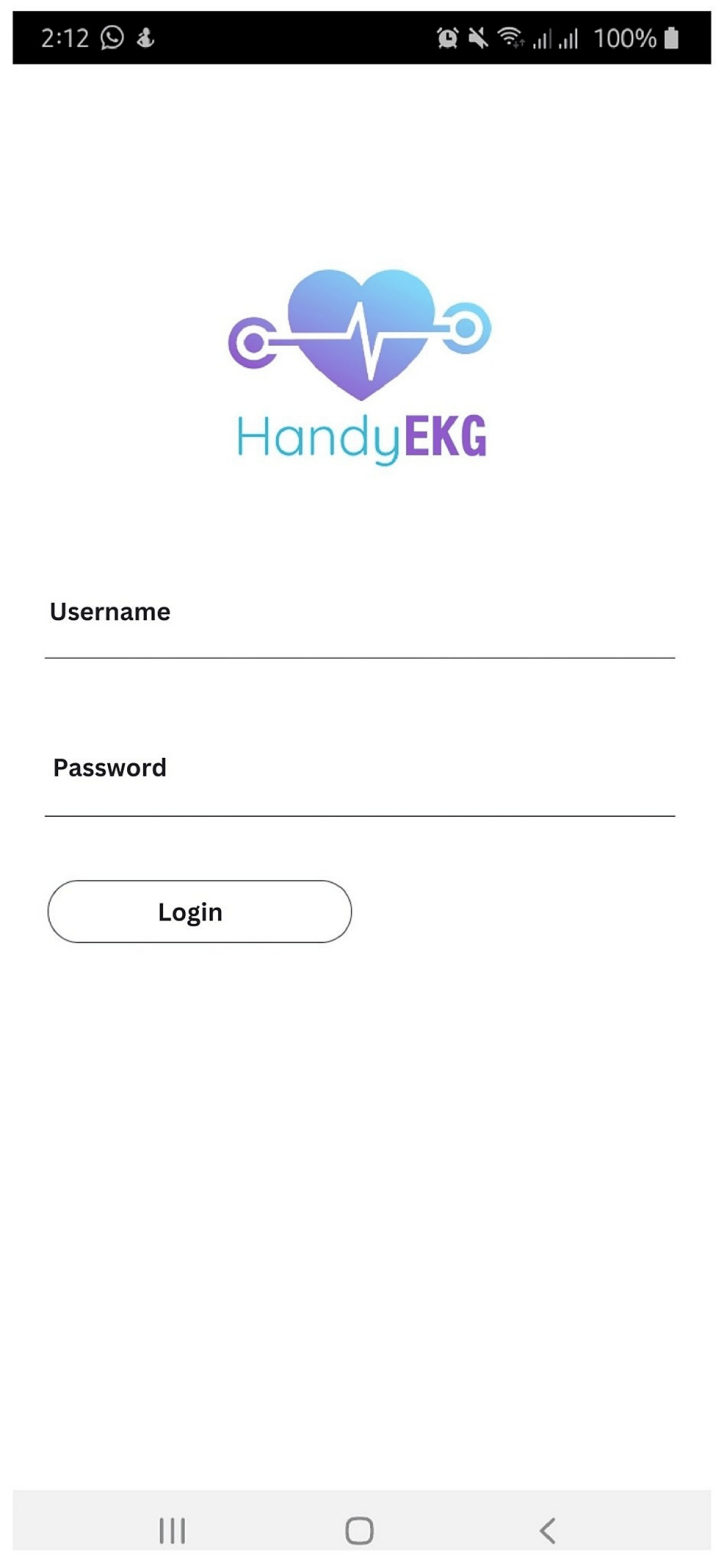
Mobile application interface.

This application (Figures 3, 4) was developed with the framework Ionic (Version 5.4.16) and Node JS (Version 12.13.0) ionic framework. This open-source front-end SDK is for developing hybrid applications based on web technologies (HTML, CSS, and JS) and PhoneGap. The application tests were developed on mobile devices with Android 9 and 10 operating systems, MinSDK: 19 Target SDK version: 27; this application is compatible with mobile devices with Android 4.4 or higher operating systems.​​​​

Phase 4. Web Platform Design

**Figure 5:**
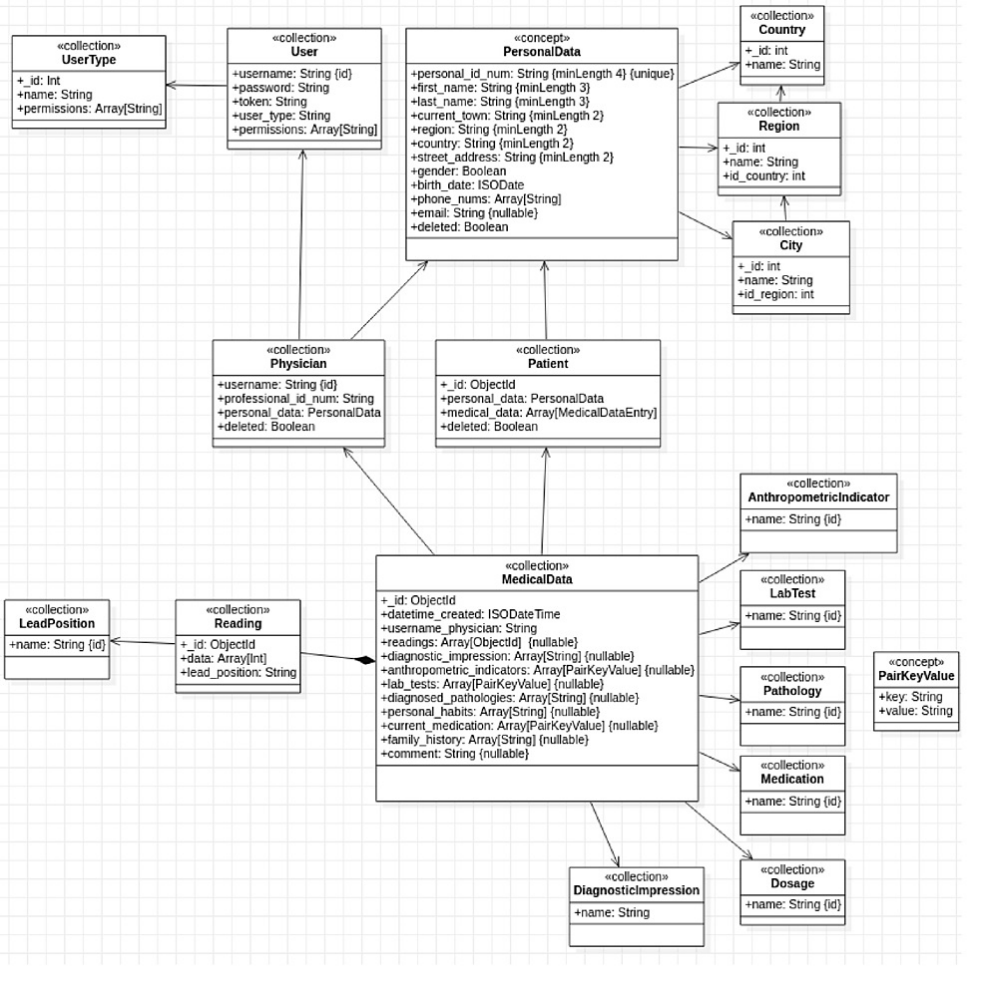
Application database diagram.

The database was designed and built (Figure 5), and the REST-type application program interface (REST API) allowed communication with the mobile application, providing access and storage of the application data from its users, patients, doctors, appointments, and readings.

**Figure 6:**
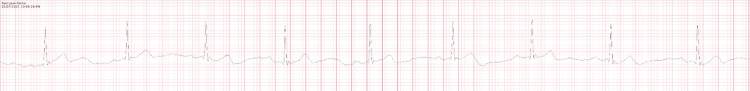
Handy EKG ECG tracing.

Ten pilot tests were performed among volunteers to identify errors in operating and communicating with the entire platform, i.e., the ECG device, web application, and mobile application. Error correction was done practically in the design elements until a prototype capable of stable reading and communication was achieved (Figure 6).

The positioning of the electrodes was determined as follows: the red electrode (negative) located in the upper right limb, the yellow electrode (positive) in the upper left limb, and the green electrode (ground pole) in any lower limb. In addition, it was possible to obtain leads II and III by alternating the positioning of the electrodes (Table 1).

**Table 1:** Positioning of the electrodes according to the derivation.

Derivation	Upper right member	Upper right member	Lower limb
I	Red electrode (-)	Yellow electrode (+)	Green electrode (N)
II	Red electrode (-)	Green electrode (N)	Yellow electrode (+)
III	Green electrode (N)	Red electrode (-)	Yellow electrode (+)

During initial testing, a comparison was made between the MIT arrhythmia database known as MIT-BIH (Massachusetts Institute of Technology Bet Israel Hospital Arrhythmia Database) [8] following the recommendations of the American National Standard for Ambulatory ECG (AAMI) [9-11]. This made it possible to find common processing stages that allowed obtaining the definitive attributes through the methodology proposed by Sannio and De Pietro [10] by following the following steps: (1) Noise reduction in the signal; (2) peak detection; (3) segmentation of the beats; and (4) temporary attribute extraction.

As a result of these stages, a new data set was obtained with which an automatic learning model was trained, specifically a deep artificial neural network that can be used to classify new readings automatically.

Validation stage

There were 51 volunteers and the demographic distribution can be seen in Table 2. We found that 7.8% (4) of the readings made with the prototype could not be assessed due to interference or noise in the signal; one of these could not be assessed with the conventional device for the same reason. The prototype was less resistant to noise than the conventional device.

**Table 2:** Volunteers demographics by gender and age.

Demographics (N = 51)	N (%)
Gender	
Female	32 (62.7%)
Male	19 (37.3%)
Age (years)	
20–29	8 (15.7%)
30–39	13 (25.5%)
40–49	14 (27.5%)
50–59	13 (25.5%)
>60	3 (5.9%)

**Figure 7:**
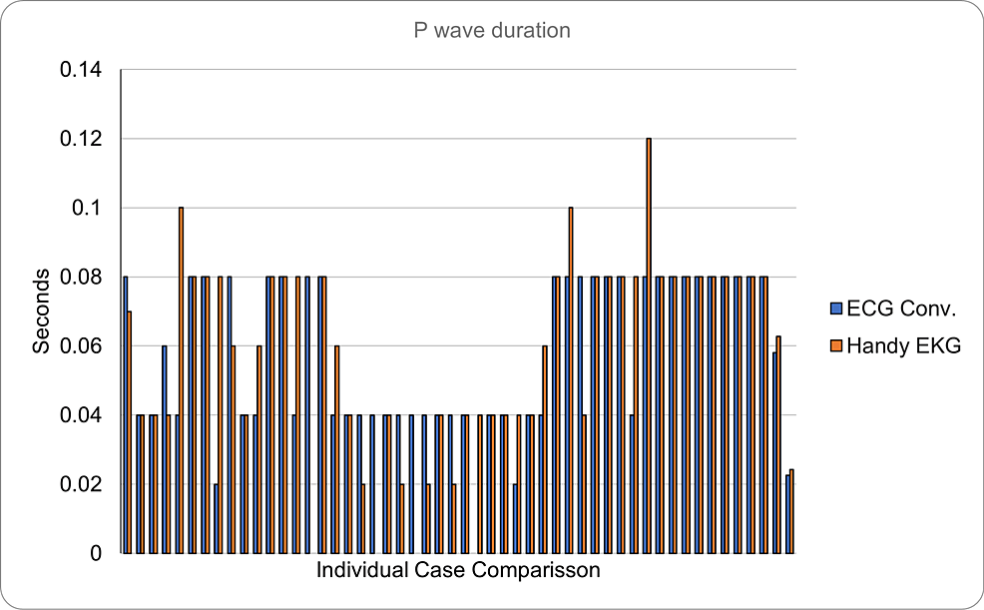
Comparison of the P wave duration between the conventional ECG and the Handy EKG. The X axis demonstrates the comparison between individual volunteers and the Y axis demonstrates the P wave duration in seconds.

**Figure 8:**
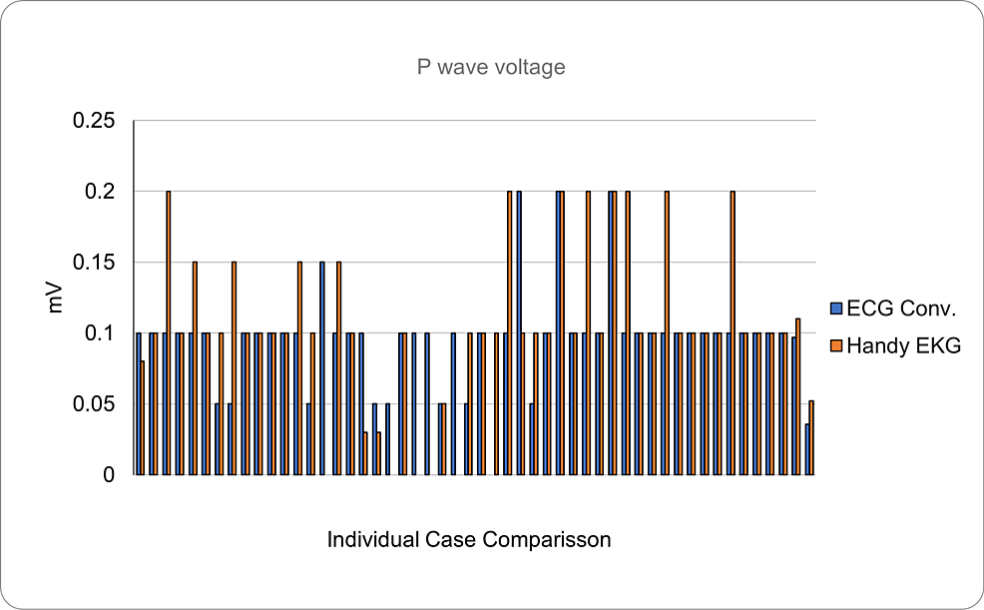
Comparison of the P wave voltage between the conventional ECG and the Handy EKG. The X axis demonstrates the comparison between individual volunteers and the Y axis demonstrates the voltage of the P wave in mV.

Regarding the P wave reading (normal duration <0.12 seconds, normal voltage <0.25 mV), it was found that the reading made with the conventional ECG had a mean duration of 0.058 seconds (SD = ±0.023) and a mean voltage of 0.095 (SD = ±0.038). In contrast, it was found that the reading made with the prototype showed a P wave with a mean duration of 0.063 seconds (SD = ±0.024) and a mean voltage of 0.108 (SD = ±0.054). Regarding the absolute error considered as the average of the differences between the measurements between both devices was 0.012 seconds for the duration and 0.035 mV for the voltage, it is important to mention that this difference, such as the following ones, also includes the human error of interpretation of the readings (Figures 7, 8).

**Figure 9:**
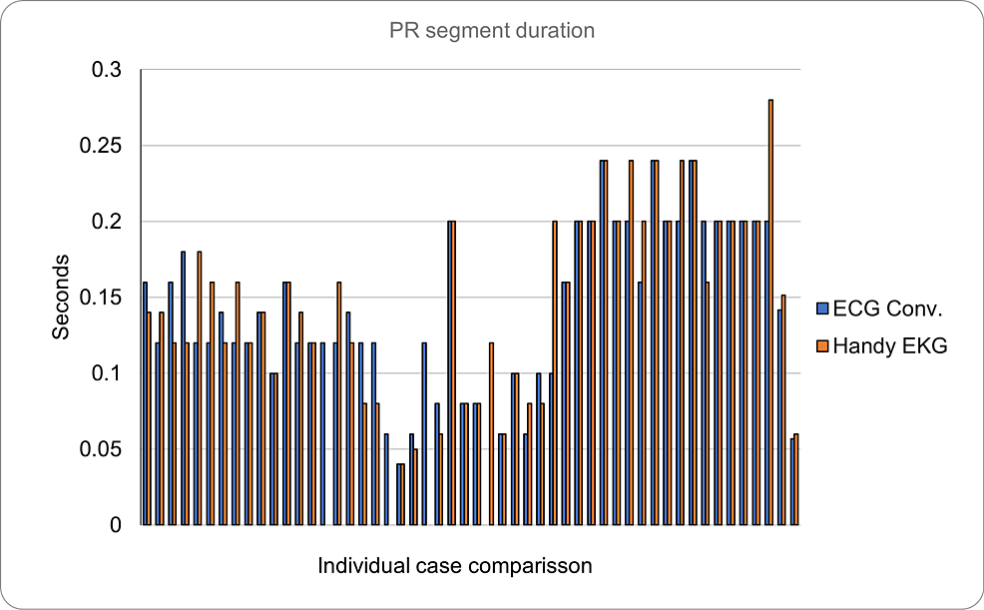
Comparison of the PR segment duration between the conventional ECG and the Handy EKG. The X axis demonstrates the comparison between individual volunteers and the Y axis demonstrates the PR segment duration in seconds.

In addition, it was found that the PR segment (normal duration 0.12-0.20 seconds) obtained in the reading made with the conventional ECG had a mean duration of 0.142 seconds (SD = ±0.057), while the reading made with the prototype showed a PR segment with duration mean of 0.151 seconds (SD = ±0.060); the absolute error between both devices for this case was 0.021 seconds. Regarding the morphology of this segment, only one reading was found to be abnormal (prolonged or shortened), and the rest were considered normal; this occurred with both devices for the same volunteer (Figure 9). Without counting the cases mentioned above, the similarity percentage from both devices was 100%.

**Figure 10:**
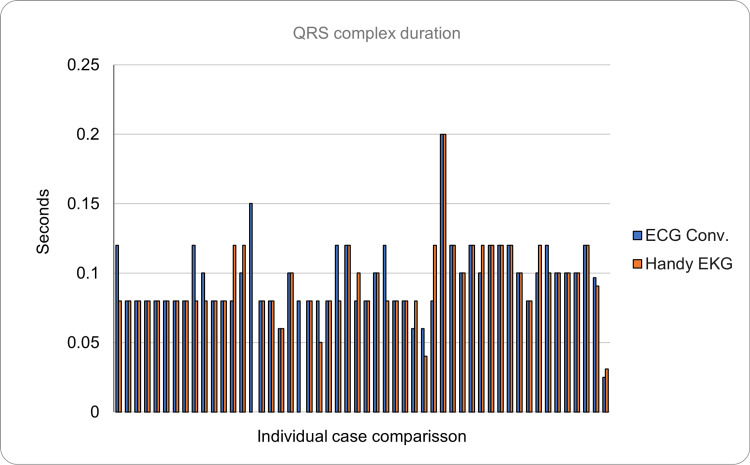
Comparison of the QRS complex duration between the conventional ECG and the Handy EKG. The X axis demonstrates the comparison between individual volunteers and the Y axis demonstrates the QRS complex duration in seconds.

The QRS complex was considered normal if all the visible waves were found and had a duration of less than 0.12 seconds. It was found that the conventional ECG reading showed a mean duration of 0.097 seconds (SD = ±0.025), while the prototype reading showed a mean duration of 0.091 seconds (SD = ±0.031). The absolute error in these measurements was 0.009 seconds. The morphology of the QRS complex was abnormal (absent Q, R, or S waves) in 18% of the readings made with the conventional ECG, while it was in 21% with the prototype. In total, there were four differences based on the evaluation of the morphology of the QRS complex between both devices, representing an equivalence of 92%. Of the readings classified as abnormal, four on the conventional ECG and six on the prototype presented with atrioventricular block Mobitz I (Figure 10).

**Figure 11:**
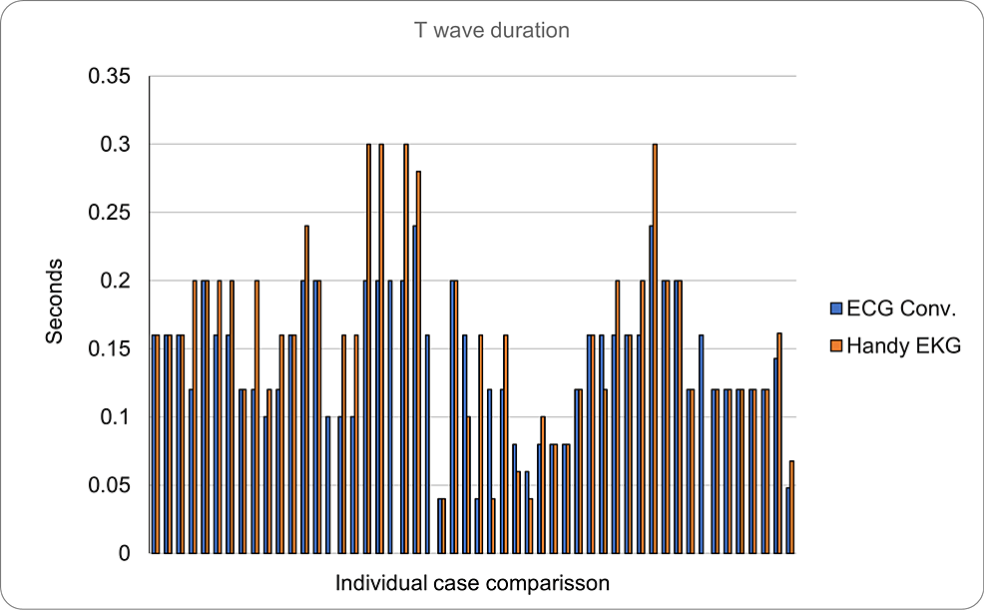
Comparison of the T wave duration between the conventional ECG and the Handy EKG. The X axis demonstrates the comparison between individual volunteers and the Y axis demonstrates the duration of the T wave in seconds.

**Figure 12:**
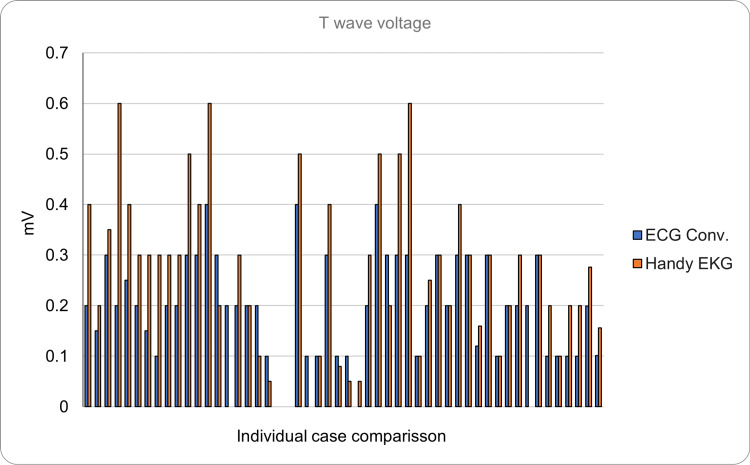
Comparison of the amplitude of the T wave between the conventional ECG and the Handy EKG. The X axis demonstrates the comparison between individual volunteers and the Y axis demonstrates the voltage of the T wave in mV.

Regarding the T wave (normal duration <0.15 seconds, voltage <0.5 mV), it had a mean duration of 0.1296 seconds (SD = ±0.0588) with a mean voltage of 0.1917 mV in the reading made with the ECG. In contrast, the reading made with the prototype showed a mean duration of 0.145 seconds (SD = ±0.0789) with a mean voltage of 0.2498 mV (SD = ±0.168). The absolute error was 0.029 seconds and 0.088 mV for the voltage. In addition to those mentioned, one more volunteer was found whose T wave could not be assessed due to noise in the signal. Figure 11 and Figure 12 show the comparison of the test results in the 51 volunteers with both devices for both duration and voltage, respectively.

**Figure 13:**
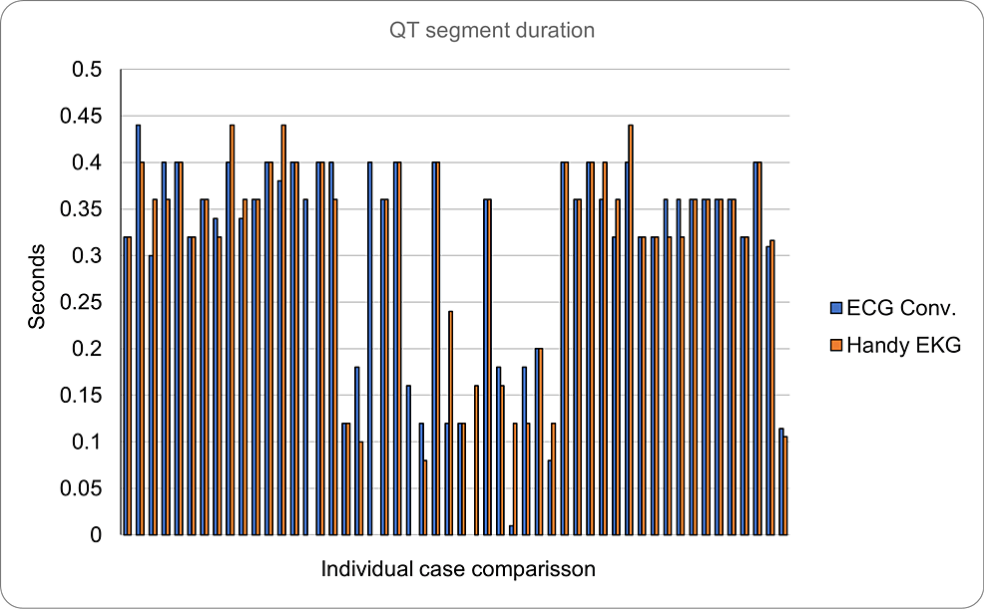
Comparison of the average duration of the QT segment between the conventional ECG and the Handy EKG. The X axis demonstrates the comparison between individual volunteers and the Y axis demonstrates the duration of the QT segment in seconds.

The ST segment (normal voltage <1 mV) had a morphology considered normal in all the readings made with the conventional ECG, and only one prototype reading was considered abnormal, so without counting the four non-assessable cases mentioned above, the diagnostic coincidence percentage between both devices for this segment was 98%. Regarding the QT segment (normal duration = <0.45 seconds), a mean duration of 0.309 seconds (SD = ±0.1114) was found in the reading made with the conventional ECG. On the other hand, the mean duration was 0.316 seconds (SD = ±0.106) in the reading made with the prototype. All the readings evaluated were considered normal in both devices, so the coincidence was 100% in this case (Figure 13).

Regarding comparing the final diagnosis (normal or abnormal) between the devices, of the 47 cases in which it was possible to assess the readings of both devices, the diagnosis coincided in 45 cases for a coincidence of 96%. In the two cases where there was a difference, the diagnosis with the conventional ECG was pathological (abnormal), while the diagnosis with the prototype was non-pathological (normal). These readings formed part of the database for the machine learning model.

Regarding the device’s safety, no health risks were identified during its use. In fact, discarding the electrodes between patients may help minimize the risk of disease transmission by dermal contact.

## Discussion

Previous similar studies have focused on describing the developmental process and included either the design of the device, the collection of medical data, or the creation of models for automatic diagnosis [12,13]. However, there are no records of studies covering the entire development process. Additionally, it is worth highlighting that various studies have considered and implemented the low-production cost approach [14-16].

This device allows continuous monitoring of patients in transit, representing an important and vital use in intrahospital care [17-19]. Although the effectiveness and efficiency of the device are indeed important, the platform represents an added practical component [20], providing possible portability and connectivity via mobile devices such as smartphones [21].

The final device provides an accurate reading of lead I of a conventional ECG, which is useful to detect brady or tachyarrhythmias. However, in the two cases where there was a difference (T wave inversion in other leads), the diagnosis with the conventional ECG was pathological (abnormal). In contrast, the diagnosis with the prototype was non-pathological (normal). The prototype has a lower capacity to detect these pathologies, mainly because it only reads one lead.

Standardized databases facilitate the interpretation and creation of these types of new technology in a developing country [22,23]. The MIT-BIH allows a precision of 98% when classifying 15 types of different arrhythmias and 63% when only classifying five types of arrhythmias. Additionally, using data analysis technologies similar to machine learning, such as deep learning techniques, among others, can favor the performance of these devices that allow an accuracy of 98% using open databases such as the MIT-BIH used in our device [24-27].

This prototype can contribute to reducing the gap in access to cardiovascular disease screening and diagnosis in the most neglected communities in Honduras. As heart diseases significantly impact global mortality, nationally disseminated methods can improve the care process for at-risk populations [28]. The cost of the prototype was estimated at around $100, which would include the device and a yearly subscription representing a possible challenge requiring critical stakeholder buy-in to reduce production costs. This would be an essential public health investment due to the global relevance of prevention strategies in health issues. As mentioned above, devices such as this offer the ability to assess other leads [29], which may help obtain a more comprehensive assessment of heart activity. However, it is necessary to emphasize that this device should not replace the conventional ECG; instead, it should be used as an adjunct in the screening and diagnosis process, which should always be confirmed by the traditional method with a conventional 12-lead ECG due to limitations that presents having only one of 15 possible derivations.

The device may contribute to creating other devices that can assess the 12 leads at a lower cost. However, obtaining lead I has a diagnostic value that should be recognized. Through this derivation, we can identify tachyarrhythmias such as atrial fibrillation [30], which represents one of the most prevalent arrhythmias worldwide. Because a large amount of data is necessary to be able to create an automatic learning model that has good predictive precision, the use of an open database and the creation of a preliminary model was proposed for this stage that in later studies counting with data labeled by cardiologist specialists can be used to create a model with the good predictive performance, which can be adapted to the epidemiological profile of the Honduran population.

One of the study’s limitations was using only healthy volunteers, which could have provided an excellent possibility for comparison and increased the sample to provide further data for the machine learning model. However, this was challenging due to the ongoing COVID-19 pandemic and the restrictions to accessing hospitals and recruiting patients in Honduras. Additionally, the reading was performed by junior doctors; nonetheless, the interpretation of basic ECG findings described in the results is considered a core competency of junior doctors.

## Conclusions

The validation stage results indicate that the designed device is safe, has good quality in its operation at all levels, and provides results that allow for diagnoses equivalent to those of a conventional ECG in most cases (considering only one lead was analyzed). The tests also showed that the prototype is more sensitive to noise (interference) than the conventional device, which made some readings not legible. However, as it is a device for medical use, the current results open the way for developing more prototypes and conducting larger-scale studies involving cardiologists and a more significant number of patients with diagnosed cardiac pathologies. This is especially necessary to provide the platform with automatic diagnostic capabilities. For this, we would have to go from a preliminary machine learning model to a production model adjusted to a database, not only with readings but with annotations made by a team of cardiology specialists who also evaluate the automatic diagnostic capacity of the platform.
